# Self-directed versus peer-supported digital self-management programmes for mental and sexual wellbeing after acquired brain injury (HOPE4ABI): protocol for a feasibility randomised controlled trial

**DOI:** 10.1186/s40814-023-01421-z

**Published:** 2023-11-29

**Authors:** Hayley Wright, Aimee Walker-Clarke, Avril Drummond, Lisa Kidd, Giles Yeates, Deborah Williams, David McWilliams, Wendy Clyne, Cain C. T. Clark, Peter Kimani, Andy Turner

**Affiliations:** 1 Centre for Intelligent Healthcare, Coventry University, CV1 5FB Coventry, England; 2 Queen’s Medical Centre, University of Nottingham, NG7 2UH Nottingham, England; 3 School of Health & Life Sciences, Glasgow Caledonian University, Glasgow, G4 0BA Scotland; 4 Rippling Minds, Oxfordshire, UK; 5 University Hospitals Coventry & Warwickshire NHS Trust, Coventry, CV2 2DX England; 6 Peninsula Medical School, University of Plymouth, PL4 8AA Plymouth, England; 7 Warwick Medical School, University of Warwick, CV4 7AL Coventry, England

**Keywords:** Brain injury, Self-management, Peer support, Sexual wellbeing, Psychosocial wellbeing, Digital health intervention, Feasibility

## Abstract

**Background:**

Acquired brain injury (ABI) can lead to biopsychosocial changes such as depression, low self-esteem and fatigue. These changes can cause, and be caused by, sexual issues affecting relationships and wellbeing. Given the relationship between sexual wellbeing and mental health, it is feasible that supporting sexual wellbeing will benefit psychological wellbeing. However, neurorehabilitation is inconsistent and often fragmented across the UK, and psychological, sexual and social support are lacking. Research shows that self-management and peer-support programmes can improve quality of life, self-efficacy and psychological wellbeing after brain injury. This protocol describes a feasibility randomised controlled trial (RCT) of a digital self-management programme to support mental and sexual wellbeing (known as HOPE4ABI), co-designed with and for people with ABI.

**Methods:**

This mixed-methods feasibility RCT has two parallel trial arms of the 8-week digital HOPE4ABI self-management programme. Eligibility criteria include age > 18 years, diagnosed or suspected ABI > 3 months prior to trial entry, access to an Internet-enabled device and ability to engage with the intervention. Referrals to the study website will be made via the National Health Service (NHS), social media and partnering organisations. Sixty eligible participants will be randomised at a ratio of 1:1 to peer-supported (*n* = 30) or self-directed (*n* = 30) HOPE4ABI programmes. Primary feasibility outcomes include recruitment and retention rates, engagement, adherence and usage. Secondary outcomes related to standardised measures of quality of life, sexual wellbeing and mental wellbeing. Participants and peer facilitators will be interviewed after the course to assess acceptability across both trial arms.

**Discussion:**

This feasibility trial data is not sufficiently powered for inferential statistical analyses but will provide evidence of the feasibility of a full RCT. Quantitative trial data will be analysed descriptively, and participant screening data representing age, ethnicity and gender will be presented as proportions at the group level. These data may indicate trends in reach to particular demographic groups that can inform future recruitment strategies to widen participation. Progression to a definitive trial will be justified if predetermined criteria are met, relating to recruitment, retention, engagement and acceptability.

**Trial registration:**

ISRCTN46988394 registered on March 1, 2023.

**Supplementary Information:**

The online version contains supplementary material available at 10.1186/s40814-023-01421-z.

## Background

Acquired brain injury (ABI) is an umbrella term referring to brain injury sustained after birth. Traumatic brain injury (TBI) and stroke account for the majority of UK ABIs (46% and 36%, respectively [1]), but other causes include brain tumour, encephalitis, meningitis and aneurysm. The UK prevalence of ABI is estimated at 2.5 million [2, 3] costing £41 billion per year [2, 4] in health and social care, lost work contributions and continuing disability [2]. ABI is a major cause of disability and disruption to families and society [5]. There are national campaigns in the UK to highlight the hidden impact and reduce the burden of ABI for patients and families [1, 6, 7], yet neurorehabilitation provision remains fragmented and inconsistent [1].

Sexual and reproductive health is integral to person-centred healthcare [8, 9] but is often neglected in neurorehabilitation [10–13]. This is commonly attributed to a lack of training opportunities and hence professional confidence and competence of healthcare practitioners [8], as well as patients’ reluctance to initiate discussions with healthcare professionals [12–14]. This results in limited opportunities for sexual (re)education and the formation and maintenance of intimate and social relationships after ABI [15].

Up to 75% of ABI survivors experience sexual problems [15, 16] totalling ~ 1.6 million people in the UK alone. Further, one in two report depression at 6 months [17], totalling ~ 1.2 million UK patients. There are complex interrelationships between neurological damage [18] and biopsychosocial changes (e.g. depression, anxiety, self-esteem [19]) following ABI. That is, biopsychosocial changes can cause—and be caused by—sexual issues [13, 20–22]. Depression has a profound impact on health and quality of life for people with ABI, resulting in more hospitalizations, less societal participation, reduced return-to-work rates, greater burden on caregivers and negative effects on social relationships [23]. Given the interrelationship between sexual health and mental health [13, 20–22], it is feasible that supporting sexual wellbeing will benefit psychological wellbeing.

Supported self-management is central to the NHS long-term plan [24] and involves professionals and patients jointly identifying needs, priorities and goals [25]. This process empowers patients with skills, knowledge and confidence to manage their own health and wellbeing. Post-stroke self-management interventions can lead to significant improvements in quality of life, self-efficacy, engagement in health-related behaviours, recovery from disability and participation in activities of daily living [26–30]. However, there are no self-management interventions for sexual wellbeing following brain injury. Likewise, peer-to-peer support approaches in neurorehabilitation have shown promising benefits, including increased behavioural control [31], self-efficacy and self-confidence; positive effects on quality of life by improving depressive symptoms, mood, psychological health and coping mechanisms; and increased knowledge, awareness and service engagement [12]. Educational resources for sex and relationships are available from many UK brain injury-related charities [10, 11, 32, 33], but lack interactive content and peer-to-peer exchange. There are currently no peer support programmes for sexual wellbeing after brain injury [12].

Patients generally attempt sexual activities 3–6 months post-ABI [16, 34], and post-3 months are, on average [35], patients’ preferred time to receive sexual information and support [36]. Digital technologies can support sexual education for patients and provide a solution to time constraints experienced by rehabilitation professionals [15]. For stigmatised topics such as sexuality, digital delivery allows autonomy, privacy and anonymity for the participant.

### Rationale

Owing to the novelty and sensitivity of the research area, it is not known whether a peer-supported intervention is an appropriate or acceptable forum for discussing sexual wellbeing after ABI. Owing to the personal nature of the topic, a self-directed intervention may be more suitable. With no comparative interventions to draw upon from research or practice, we propose a randomised controlled trial of a digital intervention, in two delivery formats: (i) peer-supported and (ii) self-directed. A feasibility RCT will address specific uncertainties, including willingness to be randomised, recruitment and retention rates, and acceptability of a sexual and mental wellbeing intervention [37–40], before conducting a definitive trial. As part of the acceptability assessment, we will also explore the appropriateness of mixed peer cohorts (e.g., whether participants would prefer peers to be of a similar age, ABI-type and/or gender).

## Methods/design

### Aim

The aim of this study is to assess the feasibility and acceptability of a digital peer-delivered intervention (HOPE4ABI) to support people with acquired brain injury to self-manage their mental and sexual wellbeing.

### Objectives

The primary objectives relate to assessing trial feasibility, via recruitment and refusal rates, retention and engagement rates for participation, and acceptability of trial procedures with a sample of participants and drop-outs for both arms of the trial. The secondary objectives will assess a preliminary signal of efficacy via pre-post change in scores on validated measures of mental wellbeing, quality of life and sexual wellbeing.

### Trial design and setting

This is a mixed methods feasibility randomised control trial, with two parallel arms: (i) peer-supported HOPE4ABI (intervention group) and (ii) self-directed HOPE4ABI without peer support (control group). The study will be conducted online with UK-based participants, hosted on a secure bespoke online research management platform, eNgage; see [41]. Participant information, consent forms and questionnaires are administered online via Qualtrics Survey Software. Examples of the participant information sheet and consent form are provided in an additional file (see Additional file 1). The digital HOPE4ABI course will be hosted by Hope for The Community (H4C) Community Interest Company, a spinout social enterprise from Coventry University [42]. Analytics data on participant use of the HOPE4ABI courses is collected routinely by the H4C platform and will be used to inform engagement and usage patterns. All data from the research platforms and software (e.g., Qualtrics, H4C) is linked by the unique participant ID within eNgage.

#### Eligibility criteria

People with any type of diagnosed or suspected acquired brain injury who meet the study inclusion criteria are eligible to participate (Table 1).

**Table 1:** Eligibility criteria for HOPE4ABI

Inclusion criteria	Exclusion criteria
• Age ≥ 18 years, UK-based • Diagnosed ABI (including head injury, stroke, meningitis, brain tumour, encephalitis, hydrocephalus, cerebral abscess, anoxic brain injury, carbon monoxide poisoning, encephalopathy, cerebral oedema, compression of the brain) ≥ 3 months prior to trial entry, category B/C/D on Patient Categorisation Tool [43, 44] ◦ OR suspected ABI, with corresponding self-reported history of brain injury, behavioural, psychological, physical, or emotional difficulties (reported at self-referral / research nurse screening phase), ≥ 3 months prior to trial entry • Capacity to give informed consent • Ability to communicate in English to participate in the intervention and complete outcome measures • Internet connection and an Internet-enabled device	• Self-reported severe mental illness (e.g. schizophrenia) • Diagnosis of dementia or other neurodegenerative disorder • Drug- or alcohol-dependency • Actively suicidal or attempted suicide in the last 3 months

#### Participant identification, recruitment and informed consent

Participants are referred to the trial through three routes: (i) self-referral, (ii) organisation referral and (iii) NHS referral, as outlined below.

#### Self-referrals

The trial will be advertised via multiple routes including social media (e.g. Facebook, Twitter), partnering organizations’ websites, newsletters and events, and the NIHR ‘Be Part of Research’ network. People with ABI may be exposed to these adverts through deliberate search (e.g. via ‘Be Part of Research’) or simply through newsfeeds on their social media channels.

#### Organisation referrals

Many partnering organisations (e.g. Headway, Brainstrust) have existing groups of research volunteers who are themselves living with brain injury. Partner organisations may distribute our trial adverts amongst their own networks of volunteers via email, social media, newsletters, meetings or events, in a more targeted approach than the self-referral described above.

#### NHS referrals

**Fig. 1:**
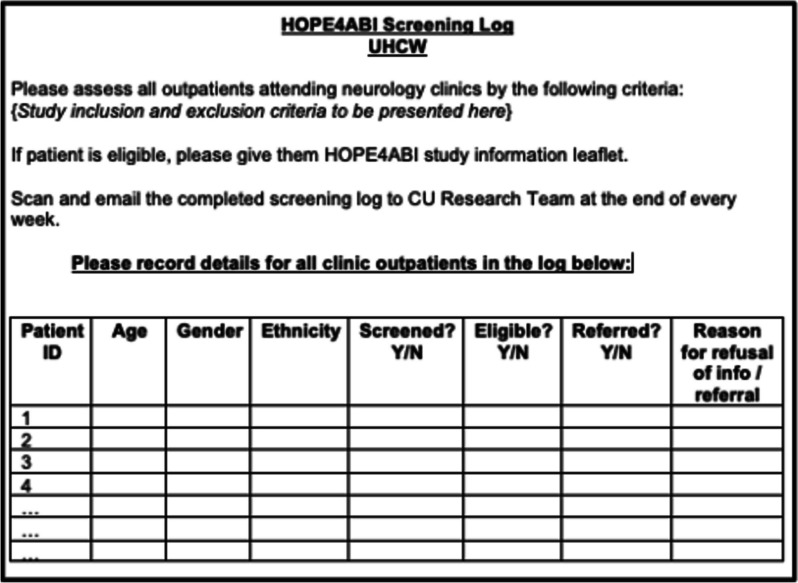
Example screening log to be completed by the clinical team at the PIC site

Participant Identification Centres (PICs) will identify eligible patients and refer them to the study website. Two trusts will initially act as PICs: University Hospitals Coventry and Warwickshire NHS Trust, and Torbay and South Devon NHS Foundation Trust. Further Trusts may be added as the study progresses (e.g. if participant recruitment rate is slow). Patients attending relevant outpatient neurology/stroke/TBI clinics will be screened against the eligibility criteria using a combination of medical notes and consultation with the clinical team. The anonymized screening log (see Fig. 1) will be completed by the clinical team and returned to the research team on a weekly basis.

**Fig. 2:**
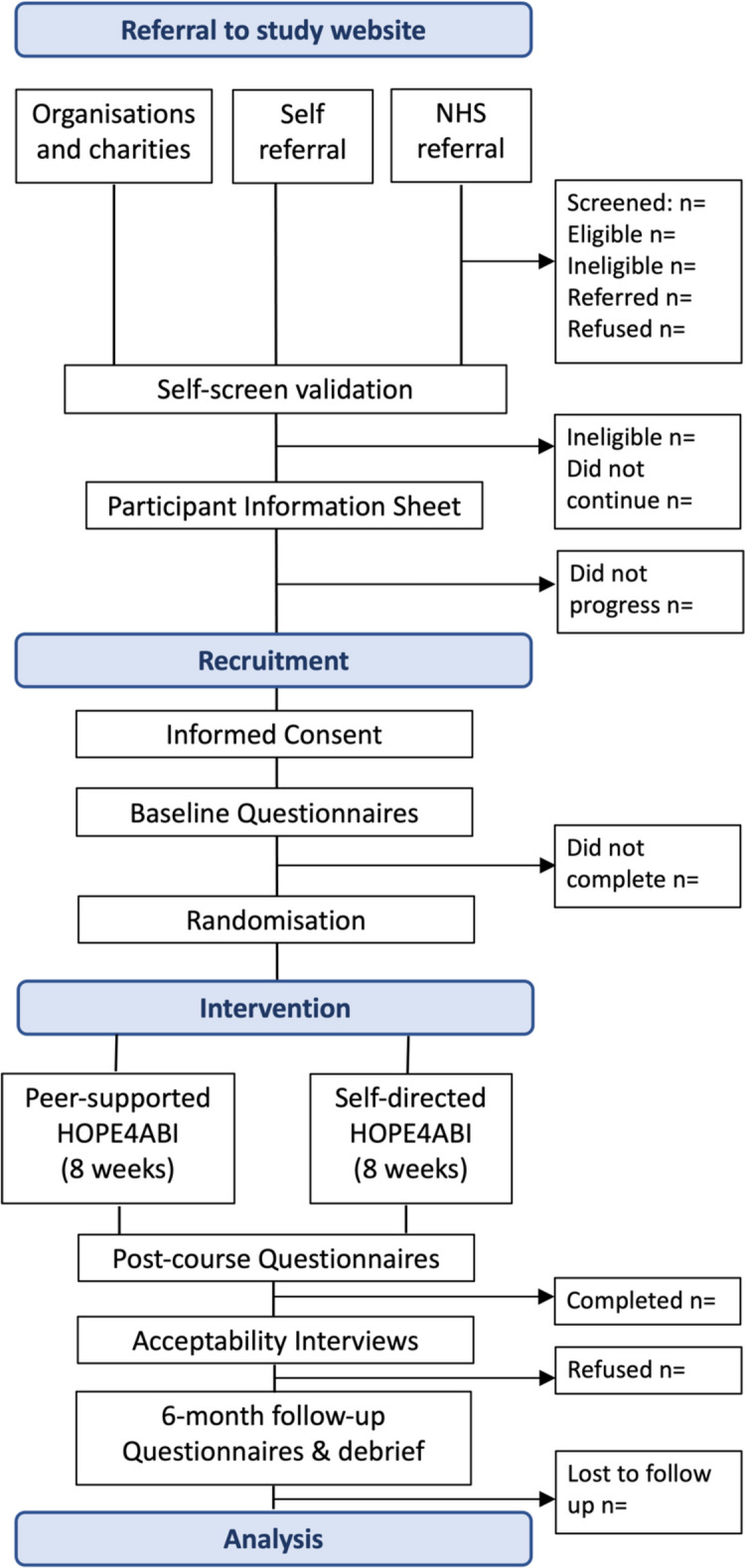
Study flow chart

In all referral routes, participants will receive information via a study advert/leaflet, containing a link/QR code to access the study website. The participants’ route through the study is summarised in Fig. 2.

After providing informed consent, and approximately 1 week before the course start date, participants complete the baseline questionnaires and are randomised to the peer-supported HOPE4ABI course (intervention group; IG) or the self-directed HOPE4ABI course (control group; CG). A subset of participants from each trial arm will be randomly selected to take part in post-course acceptability interviews. Participants who completed all or most of the intervention, as well as those who completed fewer than half of the sessions (including those who dropped out) will be interviewed to assess the acceptability of the intervention and trial procedures.

#### Randomisation, allocation concealment and blinding

The participants will be randomly assigned to the IG or CG using a 1:1 allocation ratio. Randomisation is initiated automatically on completion of the baseline questionnaires. Participants will be notified of their allocated group (IG or CG) via email, along with a weblink to join the relevant course. It will not be possible to blind participants to allocation past the point of randomisation, due to the notable differences in the intervention delivery between the IG and CG (i.e., peer-supported, or self-directed). Analysis of quantitative outcome measures will be conducted by a researcher who is blinded to allocation.

#### Interventions

HOPE4ABI will provide digital self-management support for psychological and sexual wellbeing issues that are common across different types of brain injury [10, 11]. HOPE4ABI is a novel offering, compiled of repurposed elements of an existing self-management intervention—The HOPE Programme©—alongside bespoke, co-designed sexual wellbeing support. HOPE stands for ‘Help to Overcome Problems Effectively’, and The HOPE Programme© is built on the principles of positive psychology, cognitive behaviour therapy, acceptance commitment therapy and mindfulness. It has a unique focus on hope and gratitude to create an upward spiral of positivity [45] and embeds group curative factors, such as instilling hope, universality and altruism [46], to support self-management of health and wellbeing of long-term conditions.

HOPE4ABI is an 8-week, asynchronous digital self-management programme that has been co-designed with and for people with ABI. Following a series of co-creation workshops with people affected by brain injury, common themes were identified regarding gaps in care and unmet self-management needs. Each theme is addressed across the 8-week intervention, through curated content including videos, educational content, activities, homework suggestions and additional resources (Table 2). Bespoke videos, quotes and podcasts are included featuring professionals, researchers and people living with ABI.

**Table 2:** Topics covered within each weekly module of HOPE4ABI

Session	Indicative content topics
Week 1: Instilling hope	• Introduction to self-management • The power of gratitude • Practicing self-compassion • SMARTER^a^ goal setting • *Forum/journal prompt*: reasons for joining the course
Week 2: Living with ABI	• Effects of ABI on multiple aspects of health and wellbeing • Overview of common issues caused by ABI • Talking to others about ABI • Preparing for appointments with health professionals • *Forum/journal prompt*: what are your priorities right now? What do you want to change?
Week 3: Communication	• Staying connected with others • Accepting help • Starting and managing difficult conversations • Forming deeper connections
Week 4: Sex and relationships	• Sexuality, intimacy and relationships after ABI • Embracing new norms • Understanding the needs of others • Sexual expression • New connections and dating
Week 5: Physical health	• Managing fatigue • Sleeping well • Staying active with ABI • Prioritising, planning and pacing
Week 6: Cognitive and mental wellbeing	• Understanding stress • Managing frustration • Low mood and worries • Recognising impulsivity • Brain fog—tips and tricks
Week 7: Emotional wellbeing	• Challenging unhelpful thinking styles • Building confidence and self-esteem • Mindful emotions • Self-compassion and caring for others • Reframing negative thoughts
Week 8: Living with hope	• Knowing your strengths • Happiness and hope • Building resilience • Planning meaningful activities

^a^*SMARTER* SMARTER is an acronym used by many organisations for goal setting and stands for Specific, Measurable, Achievable, Relevant, Time-bound, Enjoyable and Reward

The peer-supported HOPE4ABI course uses forums and messaging facilities that act as a conduit for communication between participants, peers and facilitators. The self-directed HOPE4ABI course contains all the same material but does not include peer support or interaction with other participants. In place of discussions, the self-directed course has a journal option for recording thoughts and making notes.

#### Safety

The peer-supported HOPE4ABI course is moderated by trained peer facilitators with lived experience of ABI. Facilitators are trained in both health coaching (provided by a QISMET accredited trainer) and sexual wellbeing coaching (provided in collaboration with The Stroke Association) and are scored by a QISMET accredited trainer throughout the course against checklists to monitor fidelity of delivery.

Mental and sexual wellbeing can be sensitive issues so participants can decide how much to engage with topics in the course. Participants who feel any emotional or psychological distress at any time can leave the activity or withdraw from the research entirely. They are advised to discuss any sexual or psychological wellbeing concerns with a professional by contacting their GP or NHS 111. If the research team suspects a participant may be at risk of harm to themselves or others, we will advise them to contact their GP, NHS 111 or call 999 (in an emergency) for further support. We will contact the participant’s GP to inform them of their patient’s participation in the study and to alert them of any welfare concerns during the trial. We may also contact emergency services on the participants’ behalf if we feel there is an immediate risk to life.

Scores on mental wellbeing measures (see *Secondary Outcomes* section for full details) will be screened at T1 (8 weeks) and T2 (6 months) for any clinically meaningful *decrease* in scores since T0 (e.g. a *reduction* in Warwick Edinburgh Mental Well-being Score of 3 or more [47]). Responses to a brief measure of mental wellbeing [48] during the intervention phase (i.e. at weeks 1, 4 and 7) will also be monitored. Participants indicating a clinically meaningful decrease in WEMWBS will be contacted by the research team and encouraged to visit their GP. Participant distress during the intervention will be managed according to the study distress protocol.

### Primary outcomes

#### Referral and recruitment phase

The number of participants who were screened, eligible/referred, recruited and refused.

#### Intervention phase

The number of participants who enrolled in the course and accessed > 50% of the course content.

#### Post-intervention (months 2–6)

The number of participants who completed follow-up questionnaires, withdrawals, drop-outs/lost to follow-up, acceptability of interventions and trial process, and intervention usage data.

### Secondary outcomes

Scores on validated questionnaires will indicate changes pre- and post-HOPE4ABI on mental wellbeing, quality of life and sexual wellbeing (summarised below). Whilst changes cannot be determined with any statistical significance in this study, we assess the feasibility of administering this set of questionnaires with a particular focus on acceptability and participant burden. Participants receive a £10 gift voucher for completing follow-up questionnaires at each of the timepoints T1 and T2. The schedule of enrolment, intervention and outcome measures is summarised in Table 3.

**Table 3:**
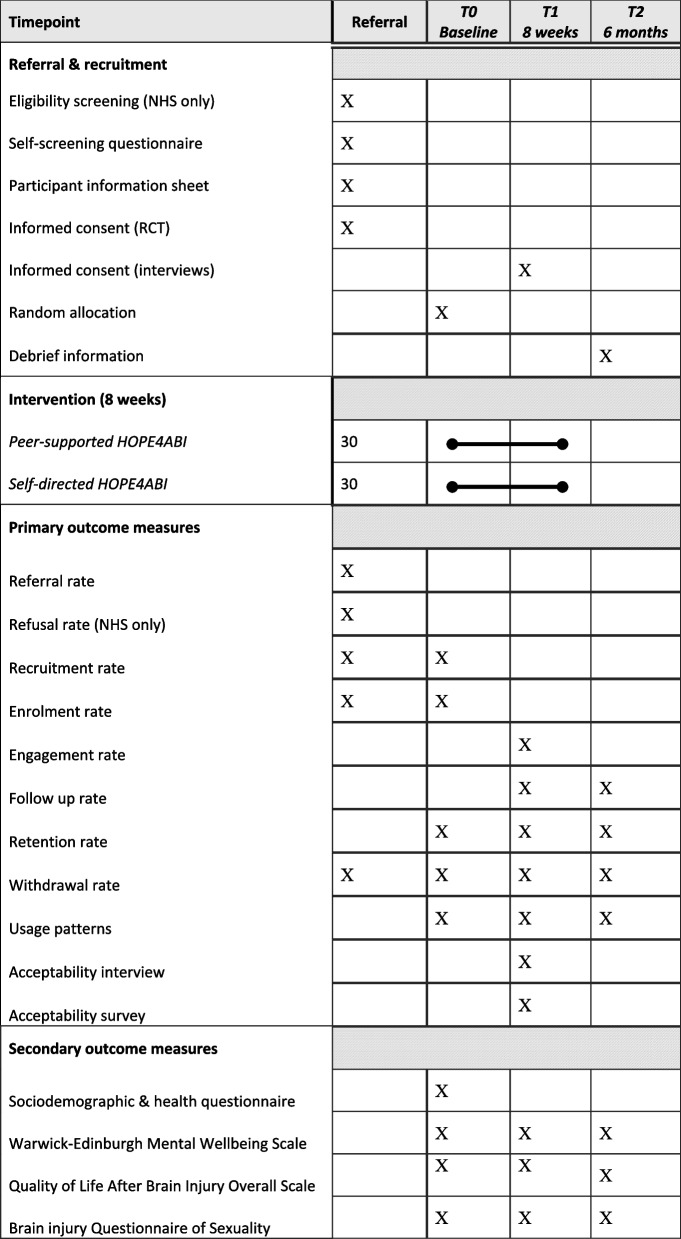
Summary of enrolment, interventions and assessments across the study period

#### Mental wellbeing

The Warwick Edinburgh Mental Wellbeing Scale (WEMWBS; [49]) is a scale of 14 positively worded feelings and thoughts, used to assess mental wellbeing within the adult population. The scale includes statements relating to experiences of positive affect, satisfying interpersonal relationships and positive functioning over the last 2 weeks. Participants rate each of the 14 items on a scale of 1 to 5, with a total positive mental wellbeing score ranging from 14 to 70, where higher scores represent greater positive mental wellbeing. A change in total score of ≥ 3 points is considered a clinically meaningful change [47].

The 7-item Short-WEMWBS (SWEMWBS [48]) will be embedded within the HOPE4ABI intervention in weeks 1, 4 and 7, so participants can monitor their own mental wellbeing throughout the course. This also allows researchers to monitor participants’ wellbeing and escalate any concerns promptly and appropriately (e.g. sudden decline in mental wellbeing score) in accordance with the study distress protocol.

#### Quality of life

Quality of Life after Brain Injury – Overall Scale (QOLIBRI-OS; [50, 51]) is a 6-item health-related quality of life measure (HRQoL) specifically tailored to patients with brain injury. QOLIBRI-OS is preferable to the full 37-item QOLIBRI [52] in this feasibility study, and since a global assessment of HRQoL is sufficient, interference from fatigue and cognitive impairment are reduced [53, 54] and participant response burden is low. Items related to satisfaction with physical, cognitive and emotional health, daily activities, social life and future prospects are scored on a 5-point scale: ‘Not at all’, ‘Slightly’, ‘Moderately’, ‘Quiet’ and ‘Very’. The total score is calculated by calculating the mean for the 6 items (provided no more than two responses are missing) and converting to a percentage by subtracting one and multiplying by 25. QOLIBRI-OS scores range from 0 to 100, with 100 being the optimal score indicating the best possible quality of life. A minimal clinically important change is a difference score of 12 [51]. QOLIBRI-OS is validated for TBI and stroke [51], meets standard psychometric criteria for reliability (Cronbach’s *α* = 0.86, test–retest reliability = 0.81) and has good construct validity in TBI populations [50].

#### Sexual wellbeing

Brain Injury Questionnaire of Sexuality (BIQS; [55]) asks participants to compare post-injury aspects of their sexuality with their pre-injury status on a 5-point Likert scale (1 = greatly decreased, to 5 = greatly increased). Fifteen questions cover changes in sexual functioning, relationship quality and self-esteem, and mood (reverse-scored). Scores across all items are summed, with higher total scores indicating more improvement. Additional questions that are not scored, provide insights such as relationship status and possible reasons for changes in sexual functioning, such as pain, fatigue and low confidence. Internal consistency (Cronbach’s *α* = 0.92), and convergent and divergent validity between the BIQS subscales and another established scale measuring sexual function [56] are good.

#### Sample size

For a feasibility trial, it is not necessary to conduct sample size calculations to power the study [57]. A randomised sample size of *n* = 60 (*n* = 30 per arm) was deemed appropriate for this feasibility study, informed by similar studies in this area [58] and National Institute of Health and Care Research (NIHR) guidelines [59]. If enrolment is < 50% (i.e. < 30 participants) halfway through the recruitment period (i.e. after 3 months), we will implement amendments to the recruitment strategy (e.g. recruiting more NHS referral sites and/or primary care settings, local and national brain injury charities and organisations). If the recruitment target is met < 3 months, the intervention period will commence ahead of schedule. For patients who have been recruited but are awaiting intervention commencement, we will send regular short updates about the study (e.g. ‘places are filling up fast’, ‘we look forward to meeting you’, ‘only 1 week to go’, etc.) via text, email, social media, etc., to maintain participant interest and prevent drop-out/attrition.

#### Data analysis

All quantitative data from the study will be analysed descriptively in concordance with the CONSORT extension for pilot and feasibility trials [60]. Sociodemographic and screening data representing the age, ethnicity and gender of all participants screened, referred or refused will be presented as proportions at the group level. This data may indicate trends in demographic groups that the HOPE4ABI study does or does not appeal to and inform future recruitment strategies to widen participation.

Measures of mean and variance, including confidence intervals and standard deviations, and number and percent for categorical variables, will be used to describe the full range of secondary outcome data (i.e. participant wellbeing questionnaire scores) at baseline and T1 and T2 follow-ups. All quantitative analyses will be performed using SPSS Statistics.

Qualitative data will be analysed by a combination of deductive and inductive thematic analysis [61], as appropriate. Interview transcripts will be read multiple times by two researchers and coded independently by each researcher. An inter-rater reliability score (Cohen κ) of < 0.70 [62] will indicate all data should be coded by a third researcher. The researchers will generate, review and refine themes and any sub-themes emerging from the data, for each of the IG and CG groups. Data derived from novel or spontaneous contributions to the interview by the participant (e.g. matter relating to acceptability not directly asked by the researcher) will be inductively coded. Qualitative data analysis may be supported using NVivo software.

#### Transition to a definitive trial

Primary outcome data (i.e. feasibility measures) will be used to examine whether progression to a definitive trial is justified, based on the following cutoffs:

1. Recruitment: ≥ 50% of eligible participants consent to take part
2. Retention: ≥ 75% of participants complete all questionnaires
3. Engagement: ≥ 75% of participants view ≥ 75% of the content in ≥ 50% (i.e. 4) modules [63, 64]
4. Acceptability: ≥ 80% of participants ‘satisfied’ or ‘very satisfied’ with HOPE4ABI delivery, content and ease-of-use

A ‘traffic light’ system will be implemented to establish progression in the following categories:

- Red (stop): i not met
- Amber (modify): i is met; AND either ii/iii/iv reach at least 70%
- Green (proceed): all criteria met

#### Data collection and management

Screening data (i.e. age, gender and ethnicity) will be recorded on a trial screening log by the research nurse/clinical team at participating NHS sites, sent to the research team on a weekly basis and transferred into a digital data file. Primary outcome data relating to feasibility measures will be collected automatically through the digital research management software (eNgage) and the digital intervention platform (H4C). Engagement and usage data will be matched to participants’ questionnaire data by the unique ID generated by the eNgage platform. Participant wellbeing data (i.e. secondary outcomes) will be collected digitally via online questionnaires administered through eNgage and Qualtrics and routinely downloaded for analysis. Acceptability interviews will be audio/video-recorded and transcribed automatically via Microsoft Teams, in accordance with the study Data Management Plan.

#### Patient and public involvement

The HOPE4ABI intervention was co-designed by people living with ABI and professionals working in ABI services, across a series of co-creation workshops. Unmet needs were explored and documented from the perspectives of patients and professionals and mapped onto self-management intervention content. Patients and professionals also took part in iterative user-testing of the HOPE4ABI intervention content prior to this feasibility RCT. A PPI representative is a joint co-applicant on the funding application for this project and has been integral to the research process from shaping the research question, developing the research design and planning a dissemination strategy.

## Discussion

HOPE4ABI is the first digital self-management intervention to support mental and sexual wellbeing after ABI. It is anticipated that this study will provide crucial evidence for the feasibility of conducting a national, multi-centre, 2 + arm, randomised controlled trial to confirm efficacy and effectiveness of HOPE4ABI for improving mental and sexual wellbeing. Through routinely collecting anonymous screening data at PIC sites, we can begin to understand any emerging trends in sample bias, for example, relating to age group, ethnic background or gender. This data will be used to inform recruitment strategies and accessibility issues in any future trials, to widen participation to diverse participant groups. Issues pertaining to equality, diversity and inclusion are more important than ever in healthcare, so addressing these from the outset is imperative to improving access to, and benefit from, services to all people. Progression to a definitive trial will be justified if predetermined criteria are met, relating to recruitment, retention, engagement and acceptability.

### Trial status

At the time of the first protocol submission (July 2023), the trial had not yet started recruiting. At the time of the revised protocol re-submission (October 2023), the intervention phase was underway. Seventy-two participants consented to take part, and 53 completed baseline measures and were randomised to the intervention (*n* = 27) and control (*n* = 26) arms.

### Supplementary Information

**Additional file 1.** Examples of the participant information sheet and consent form.

## Data Availability

The anonymised datasets created from the current feasibility study may be available from the corresponding author on reasonable request.

## Abbreviations

ABI: Acquired brain injury
BIQS: Brain Injury Questionnaire of Sexuality
CG: Control group
CONSORT: Consolidated Standards of Reporting Trials
GP: General practitioner
HCRW: Health and Care Research Wales
HRA: Health Regulations Authority
HRQoL: Health-related quality of life
H4C: Hope For The Community, Community Interest Company
IG: Intervention group
IRAS: Integrated Research Application System
ISRCTN: International Standard Randomised Controlled Trial Number
NHS: National Health Service
NIHR: National Institute for Health and Care Research
PI: Principal investigator
PIC: Participant Identification Centre
PIS: Participant information sheet
PPI: Patient and public involvement
QISMET: Quality Institute for Self-Management Education and Training
QOLIBRI-OS: Quality of Life after Brain Injury—Overall Scale
RCT: Randomised controlled trial
REC: Research ethics committee
RfPB: Research for Patient Benefit Programme
R&D: Research and development
SD: Standard deviation
SWEMWBS: Short Warwick-Edinburgh Mental Wellbeing Scale
TBI: Traumatic brain injury
UHCW: University Hospitals Coventry & Warwickshire
WEMWBS: Warwick-Edinburgh Mental Wellbeing Scale
WHO: World Health Organization
