# Supplementary material for: Self-directed versus peer-supported digital self-management programmes for mental and sexual wellbeing after acquired brain injury (HOPE4ABI): protocol for a feasibility randomised controlled trial
Source: Pilot Feasibility Stud. 2023 Nov 29;9:194. doi: 10.1186/s40814-023-01421-z (PMC10685616; doi:10.1186/s40814-023-01421-z)
Supplement: Supplementary file 1 — Additional file 1. Examples of the participant information sheet and consent form. [file 40814_2023_1421_MOESM1_ESM.pdf]

Digital self-management for mental and sexual wellbeing after acquired brain injury (HOPE4ABI): feasibility randomised controlled trial

IRAS Project ID: 325598

PARTICIPANT INFORMATION SHEET

We are inviting you to take part in a research study to test a new 8-week online course for people living with acquired brain injury (ABI). Before you decide to take part, it is important you understand why we are doing the research and what it will involve. Please read this information carefully and talk about it with family or friends if you want to.

What is the purpose of the study?

ABI affects people in different ways. Rehabilitation tends to focus on physical and cognitive health – for example, walking and talking. Mental health, sexual wellbeing and relationship problems are often neglected. These issues can be overwhelming and affect other areas of life.

Together with people living with ABI, we designed an online course to support mental and sexual wellbeing (called Hope for ABI, or 'HOPE4ABI'). The purpose of this study is to test HOPE4ABI in randomised controlled trial, or RCT.

Important note: this study is not meant to replace psychological therapy or medical treatment. We aim to help you to manage your own mental and sexual wellbeing. Please contact your GP if you think you need professional advice or support.

Why have I been chosen?

We have invited you because you are over 18 years old, and you had an ABI at least 3 months ago. We would like you to take part in the new HOPE4ABI intervention and tell us how you got on afterwards. To take part in the study, you will need access to an internet-enabled device, such as a smartphone, laptop or tablet.

What are the benefits of taking part?

The HOPE4ABI course may help you to manage your own mental and sexual wellbeing. You may meet new friends, or learn new skills, or find out something new about yourself.

We will email you a link to questionnaires that measure your wellbeing before the course begins. We will send you a £10 gift voucher each time you complete the follow up questionnaires – once after the course, and then again after 6 months.

Are there any risks associated with taking part?

The study is online and there is little physical risk of taking part. However, mental and sexual wellbeing can be sensitive issues. You can decide how much to participate in topics that you

might find uncomfortable. If you feel emotional or psychological distress at any time, you are welcome to leave the activity, or withdraw from the research entirely. If you want to discuss your sexual or psychological wellbeing with a professional, please contact your GP, or call NHS 111. Whatever you're going through, there are people you can talk to any time. If you need immediate support, you can:

- call **Samaritans** on **116 123** (UK-wide) - A 24/7 service for you to talk through anything which may be causing you distress at this time
- text **SHOUT** to **85258** (UK-wide) – A 24/7 confidential service, free to text from all major mobile networks in the UK

If we suspect that you may be at risk of harm to yourself or others, we will advise you to contact your GP, NHS 111, or call 999 (in an emergency). We will also write to your GP to inform them, and we may contact emergency services on your behalf if we feel there is an immediate risk to life. If you tell us you have engaged in criminal activity, we will report this to Coventry University Ethics Committee. They will decide on further action and may refer the matter to the police.

Do I have to take part?
No – it is your choice. If you are unsure, please take time to discuss with your family or friends or ask the researchers if you have any questions.

How do I withdraw from the study?
You can withdraw your participation from the study at any time. You do not need to give a reason, and your rights will not be affected. Just let the research team know, using the contact details on the study website.

What will happen if I decide to take part?
There is a digital consent form on the next page. If you decide to take part, you will need to confirm all the statements to show that you understand your rights and are happy to participate. The diagram below shows the steps you will take through the rest of the study.

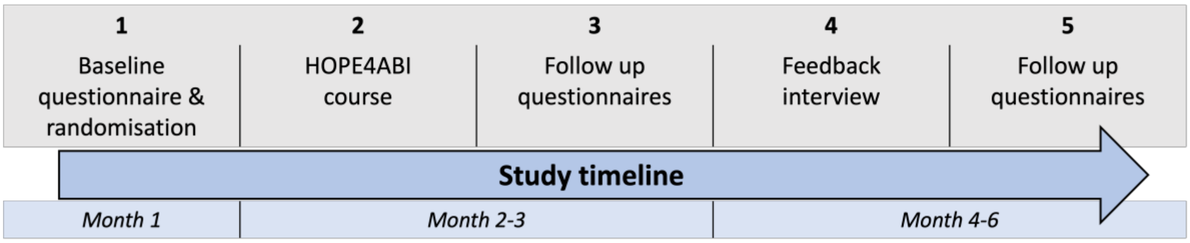

1. You will be asked to fill in baseline questionnaires to tell us all about you, including contact details, education, marital status, and some details about your ABI. You will only need to complete this once.

   Another set of short questionnaires will measure your mental wellbeing, quality of life, and sexual wellbeing. This will take you around 15-20 minutes. You will complete these again at the two follow up points so we can compare your answers before and after the course.

Next, the computer will assign you to either: **peer supported** HOPE4ABI, or **self-directed** HOPE4ABI. This is decided at random, like the flip of a coin. You have a 50/50 chance of being put in any group.

2. The 8-week HOPE4ABI course contains a range of self-management tools, tips, and activities. Different formats such as videos, podcasts, and quizzes will be used to cover topics relating to psychological and emotional wellbeing, intimacy, and personal and social relationships. A different topic will be covered each week, with the content being released at the same time every week, e.g. 11am on a Wednesday. Each week's content will take around 1 hour, but you can work through it at your own pace across the week. All aspects of the course are **optional**.

   The **peer-supported** course will offer options for you to interact with other participants (i.e., your peers) and will be guided by a peer 'facilitator' who will oversee your progress and be on hand to answer questions. The **self-directed** course has the same topics and content, but you will have no contact with other participants and there will be no facilitator.

3. A week after your HOPE4ABI course has ended, we will email you a link to the wellbeing questionnaires, plus a short feedback questionnaire about your experience on the course. These will take you around 15-20 minutes to complete. We will email you a £10 gift voucher as a thank you for completing them.

4. We will invite participants at random to tell us in more detail about your thoughts, opinions, experiences of the study. Interviews will take place on Microsoft Teams at a time that suits you and will last around 30 minutes. The interview will be recorded and automatically transcribed by the Microsoft Teams software. These will be checked for accuracy by a member of the research team after the interview, any identifying information will be removed from the transcription, and then the video recording will be deleted. Your opinions will help us to improve the course for future participants.

   If you are randomly selected for an interview, we will send you an invitation after the course. You do not have to decide now, and you do not have to say yes. We will send you the relevant information to help you decide at the time.

5. After six months, we will email you a link to the same **wellbeing** questionnaires, plus a few questions about what you learned on the course. These will take you around 15-20 minutes to complete. We will email you another £10 gift voucher as a thank you for completing them. This six-month follow up marks the end of the study.

How will we use information about you?
We will need to use information from you for this research project. This information will include your:
- Initials
- Participant identification number
- Internet Protocol (IP) address
- First name and surname

- Date of birth
- Gender identity
- Ethnicity
- Postcode
- Contact details (home address, email address, telephone number)
- GP contact details (name of GP, practice address)
- Highest level of education
- Employment status
- Relationship status
- Living arrangements (i.e. alone or with others)
- Sexual orientation
- Type of brain injury and number of years since it happened
- Answers given on questionnaires to measure your psychological and sexual wellbeing, and quality of life

We will use this information to do the research. People who do not need to know who you are will not be able to see your name or contact details. Your data will have a code number instead. We will keep all information about you safe and secure. **On the HOPE4ABI course, you can use a made-up name for yourself if you want to remain anonymous to other participants.**

Once we have finished the study, we will keep some of the data so we can check the results. We will write our reports in a way that no-one can work out that you took part in the study.

What are your choices about how your information is used?

- You can stop being part of the study at any time, without giving a reason, but we will keep information about you that we already have.
- We need to manage your records in specific ways for the research to be reliable. This means that we won't be able to let you see or change the data we hold about you.
- If you agree to take part in this study, you will have the option to take part in future research using your data saved from this study.
- Identifiable data (e.g. name, contact details) will be destroyed at the end of the study period **31/10/2024**. Your anonymised data (e.g. questionnaire responses with names removed) will be kept in long-term digital storage for up to 10 years after the trial has ended and will be permanently deleted by **31/10/2034**.

Where can you find out more about how your information is used?

You can find out more about how we use your information:

- at www.hra.nhs.uk/information-about-patients/
- our leaflet available from www.hra.nhs.uk/patientdataandresearch
- by sending an email to Coventry University Data Protection Officer at enquiry.igu@coventry.ac.uk

What will happen with the results of this study?

This study will help us to make sure the research process is suitable, accessible, and appropriate for people with ABI. Next, we aim to run a large-scale RCT to evaluate the effect of HOPE4ABI on mental and sexual wellbeing in a larger group of people with ABI.

We will report results of this study in journal articles, research reports, newsletters, blogs, and presentations. Quotes or key findings will always be made anonymous for reporting purposes.

We will email you a summary of the study results at the following timepoints:
- After the 8-week course has finished and questionnaires have been completed
- After the 6-month follow up questionnaires have been completed
- After the final study results are published (Spring 2025).

What if something goes wrong?
Coventry University agrees that in respect of any personal injury or death of any participant as a result of participation in the study, it will provide no-fault compensation and will be insured to pay out on any such claims.
If you are unhappy with any aspect of this research, please contact the research team:
Dr Hayley Wright - ab7764@coventry.ac.uk Office Tel: 02477 651413
Prof Andy Turner – hsx116@coventry.ac.uk

You can also report any concerns to the Patient Advice and Liaison Service, a free and confidential service for patients and their families:

Post: Patient Advice and Liaison Service, University Hospital Coventry & Warwickshire NHS Trust, Clifford Bridge Road, Coventry CV2 2DX.
Email: feedback@uhcw.nhs.uk (Start your subject line with FAO: PALS)
In person: At the PALS Centre at University Hospital, Coventry; or by request at main reception at the Hospital of St. Cross, Rugby.

Monday to Friday: 8am-8pm; Saturday: 12pm-5:30pm; Sunday: 1pm-5:30pm

Freephone: 0800 028 4203 (24 hour voicemail available)

Trust Website: www.uhcw.nhs.uk

If you still have concerns or wish to make a formal complaint, please write to:
Coventry University Ethics Committee, Coventry University, Coventry CV1 5FB; or email ethics.uni@coventry.ac.uk. In your letter please provide information about the research project, give the names of the researchers, and detail the nature of your complaint.

Digital self-management for mental and sexual wellbeing after acquired brain injury (HOPE4ABI): feasibility randomised controlled trial
<u>**IRAS Project ID: 325598**</u>

INFORMED CONSENT FORM

You are invited to take part in this research study to test a new online course for people with ABI. Please contact us (ab7764@coventry.ac.uk; Office Tel: 02477 651413) if anything is unclear or if you would like more information.

If you are happy to take part, please confirm your consent by **typing your initials** against each of the below statements:

	YES	NO
I confirm that I have read and understood the Participant Information Sheet (v2 30/04/2023) for the above study and have had the opportunity to ask questions		
I understand that my participation is voluntary and that I am free to withdraw at any time without giving a reason, without my rights being affected		
I understand that my identifiable data will be deleted at the end of the study (**31/10/2024)**		
I understand that anonymised data will be kept in long term digital storage for up to 10 years after the study has ended, and will be deleted by 31/10/2034		
I understand that all the information I provide will be held securely and treated confidentially		
I am happy for my GP to be informed of my participation in the study		
I understand that the research team may contact my GP if they feel that I pose a risk of harm to myself or others		
I am happy for the information I provide, including direct quotes, to be used (anonymously) in academic papers and other formal research outputs		
I am happy to be invited to take part in an interview, if I am randomly selected [optional]		
I am happy to be contacted about options to take part in future studies [optional]		
I agree to take part in the above study		
